# Clinical and radiological outcome of surgical hallux valgus correction: open versus minimally invasive

**DOI:** 10.1007/s00590-024-04074-7

**Published:** 2024-08-20

**Authors:** Andreas Pappas, Alexander Hönning, Marc D. Schmittner, Axel Ekkernkamp, Denis Gümbel

**Affiliations:** 1 Department of Orthopaedics and Trauma Surgery, Havelland Clinic Nauen, Ketzinerstr. 21, 14641 Nauen, Germany; 2 Centre for Clinical Research, BG Klinikum Unfallkrankenhaus Berlin gGmbH, Berlin, Germany; 3 Department of Anaesthesiology, Intensive Care and Pain Medicine, BG Klinikum Unfallkrankenhaus Berlin gGmbH, Warener Str. 7, 12683 Berlin, Germany; 4 Medical Faculty Mannheim of Heidelberg University, Ruprecht‐Karls‐University Heidelberg, Ludolf-Krehl-Straße 13-17, 68167 Mannheim, Germany; 5 Department of Trauma and Orthopaedic Surgery, BG Klinikum Unfallkrankenhaus Berlin gGmbH, Warener Str. 7, 12683 Berlin, Germany; 6 Department of Trauma, Reconstructive Surgery and Rehabilitation Medicine, University Medicine Greifswald, Ferdinand-Sauerbruch-Straße, 17475 Greifswald, Germany

**Keywords:** Hallux valgus, Minimally invasive, Outcome, Intermetatarsal angle, Pain, Complication

## Abstract

**Introduction:**

Hallux valgus (HV) is the most common forefoot deformity. Surgical correction of HV aims to reduce pain, preserve joints, and re-establish foot function while restoring the hallux valgus angle (HVA) and intermetatarsal angle (IMA). Many surgical procedures have been proposed, including open and minimally invasive (MI) techniques. This study aimed to compare the midterm outcomes of open vs. MI procedures and their impact on the duration of surgery, hospital stay, HVA, and IMA post-operatively.

**Materials and methods:**

One hundred and twenty HV patients operated by open or MI surgery between October 2019 and October 2022 were included. One hundred three patients met the inclusion criteria and consented to the study. Patients were prospectively surveyed for foot functionality, post-operative pain, and complications using the AOFAS score. Radiographic measurements of HV angles, length of hospital stay, and surgery duration were analysed.

**Results:**

MI surgery patients had significantly better AOFAS scores (*p* < 0.001) 12 months post-operatively compared to open surgery. Complication rates were lower in the MI group (3.8% vs. 33.3%, *p* < 0.001). MI surgery patients also had shorter hospital stays (0.9 ± 0.3 days vs. 2.0 ± 0.0 days) and surgery duration (19.7 ± 2.3 min vs. 80.7 ± 6.8 min). MI surgery was more effective in correcting the IMA but equally effective as open surgery for HVA correction.

**Conclusion:**

MI surgery resulted in better patient satisfaction, fewer complications, and more precise correction of IMA values. Moreover, the duration of surgery and hospital stay were significantly lower in patients undergoing MI surgery. Further research is needed to validate these findings in controlled, prospective randomised trials.

## Introduction

Hallux valgus (HV), commonly known as a bunion, has a global prevalence of 19–35% and primarily affects women aged 50–70 [1–4]. It is characterised by the progressive abduction and pronation of the first phalanx and the adduction, pronation, and elevation of the first metatarsal (MT). HV can cause significant metatarsalgia [5, 6].

Diagnoses are primarily clinical, supported by radiographic measurements of the hallux valgus angle (HVA) and the intermetatarsal angle (IMA), which allow classification of the deformity severity.

Many surgical and nonsurgical treatments for HV have been proposed, but outcomes vary regarding foot function, pain, and complication rates [7–11].

This study aims to compare the midterm outcomes of open surgical techniques (e.g. Chevron-Austin, Lapidus, and Scarf osteotomies) versus a third-generation minimally invasive proximal osteotomy with bicortical screw fixation.

## Materials and methods

### Study design

The present study is a retrospective, single-centre, descriptive observational study with prospectively collected post-operative patient data. It compares the effectiveness and safety of minimally invasive and open surgical procedures for HV correction. One hundred and twenty patients treated between October 2019 and October 2022 met the eligibility criteria, of which 103 (i.e. 14.2% drop-out rate) completed the follow-up examination. This trial was not randomised; treatment decisions were based on surgeon expertise, patient preference, and specific clinical indications. Once HV deformity was diagnosed and surgery was indicated, a decision on MI or open surgery was made based on the preferred method by the patients.

### Inclusion and exclusion criteria

Inclusion criteria

- Clinical diagnosis of HV surgical indication
- Age > 18 years
- Consent to participate

Exclusion Criteria

- Severe deformities requiring more extensive surgical procedures (i.e. claw toe, hammer tow)
- Presence of polyneuropathy or other medical conditions affecting surgical outcomes
- Pregnancy or lactation
- Allergies to materials and medications used intraoperatively and post-operatively

**Fig. 1:**
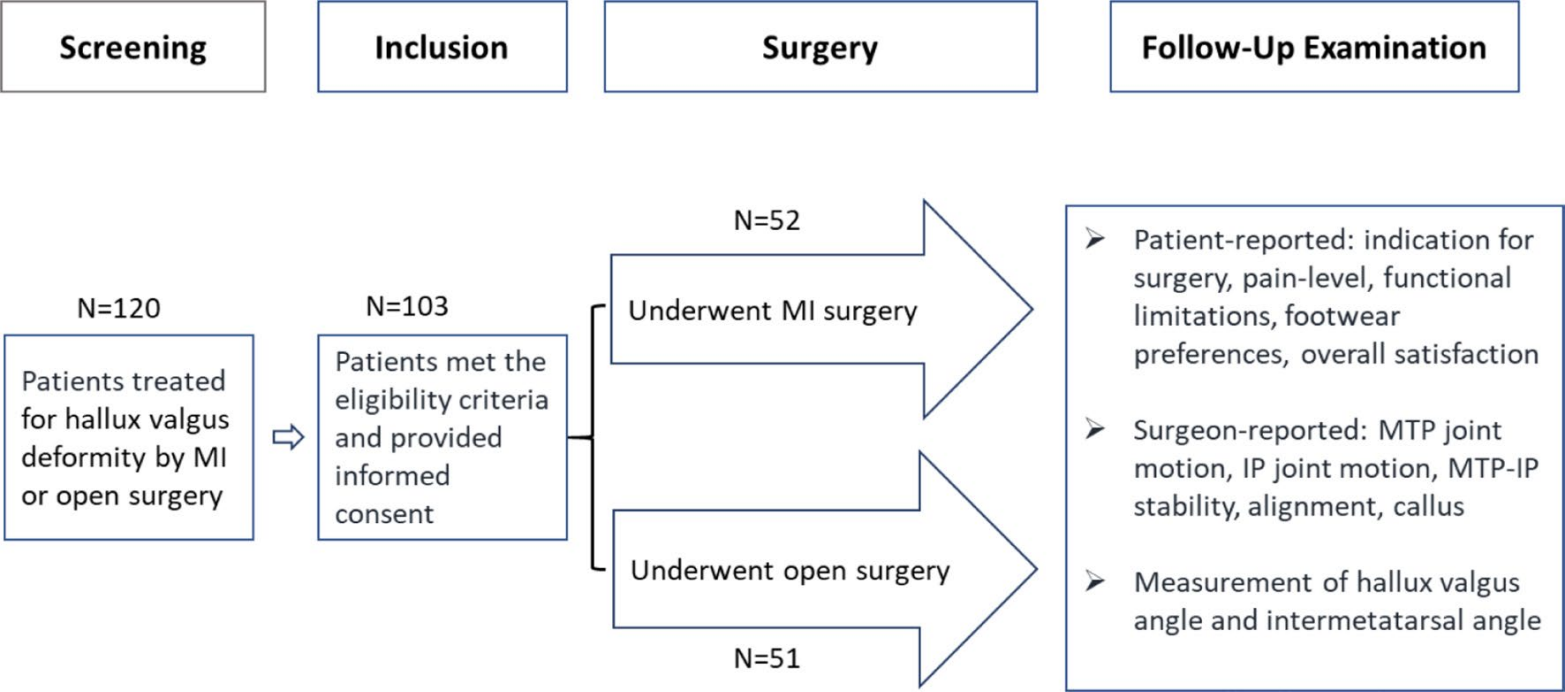
Study flow chart

Foot functionality was assessed using the standardised AOFAS score before and after the surgery. A total of 51 patients underwent the open surgical procedure, while 52 patients received the minimally invasive treatment (see also the study flow chart in Fig. 1).

Before commencing data collection, the study was submitted to and approved by the relevant ethics committee (Reference Number: Eth—22/2022). Care was taken to ensure that data collection and processing complied with data protection regulations.

### Surgical procedures

MI and open procedures followed standard protocols with general anaesthesia or nerve blocks and prophylactic antibiotics. The patient was positioned on the operating table in a supine position; subsequently, the foot region was prepped and draped in the usual sterile fashion.

In open surgery, an incision was made along the lateral side of the first metatarsal–phalangeal joint (MTPJ). The underlying soft tissues were carefully prepared, and the abductor hallucis muscle was gently retracted. The first step was the arthrotomy with proximal exostosis osteotomy of the first metatarsal bone (MTP-I). Depending on the method chosen, an osteotomy of the first metatarsal bone (MTP-I) was performed (Austin operation). The osteotomy was aligned to correct the IMA. The osteotomy was then stabilised using a sturdy plate and screws, ensuring correct bone positioning. Finally, the soft tissues were meticulously closed, and a sterile dressing was applied to protect the wound.

**Fig. 2:**
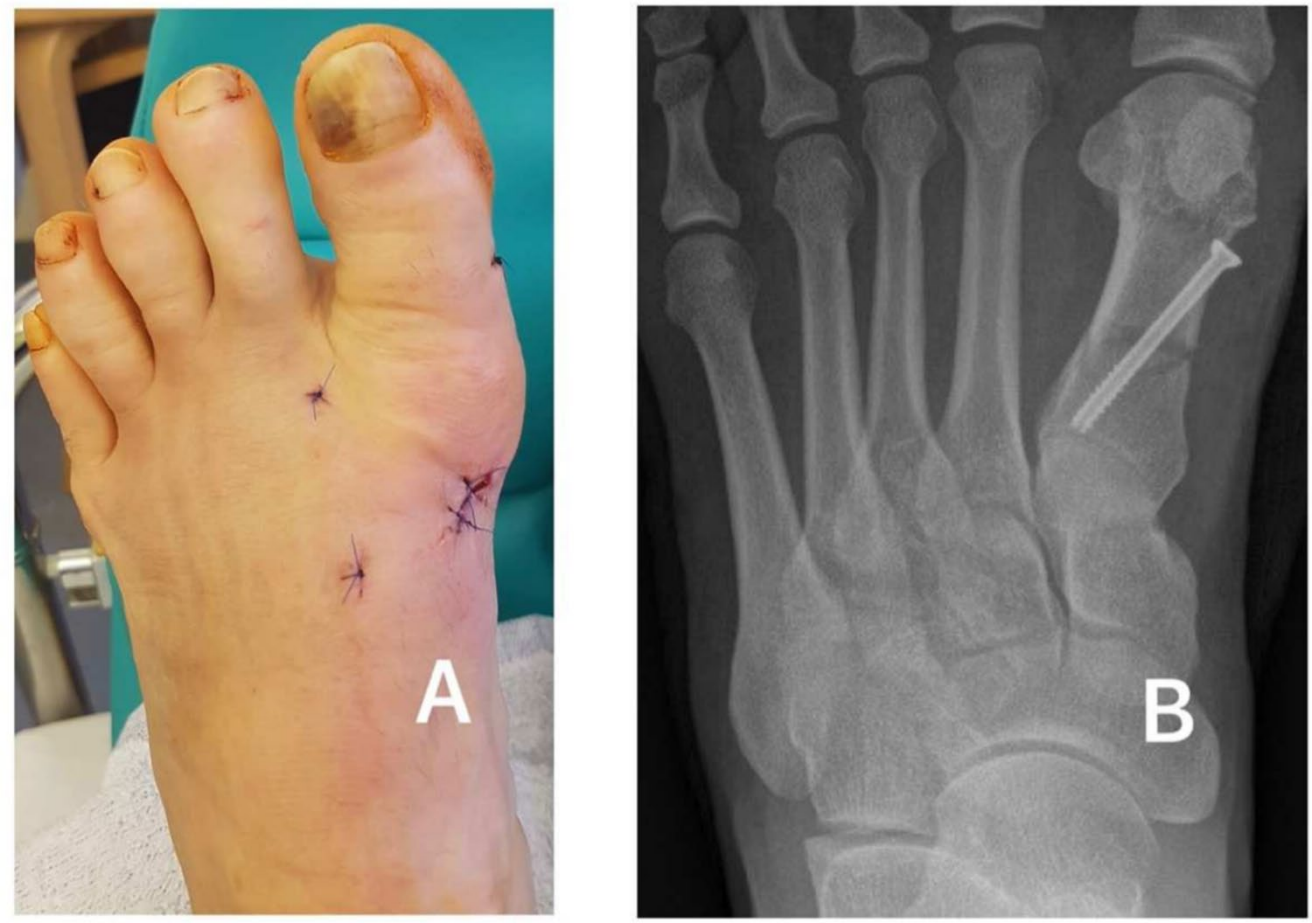
A: Post-operative clinical image, **A** and X-ray, **B** after minimally invasive proximal osteotomy and screw fixation

**Fig. 3:**
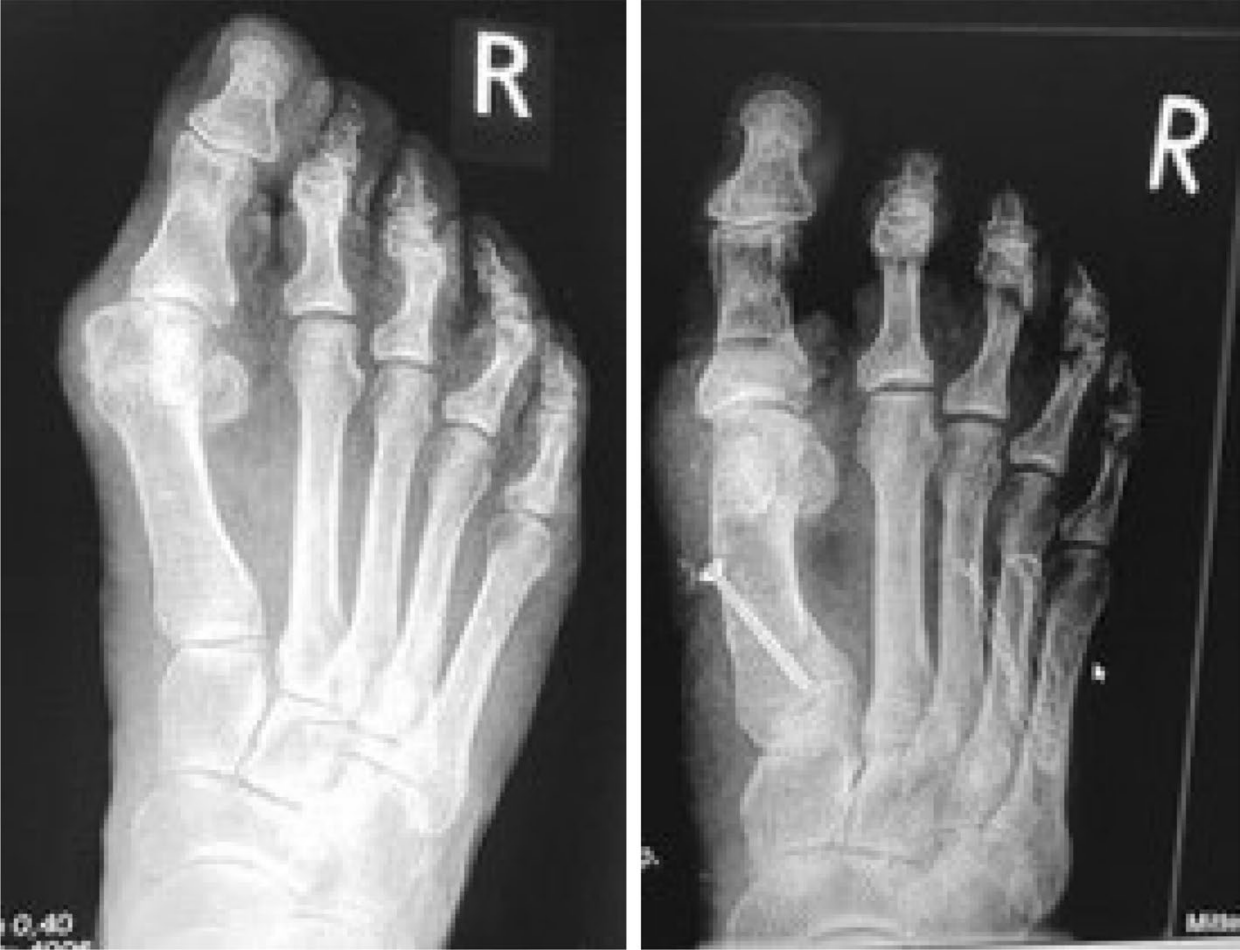
Example of pre- and post-operative X-rays following MI surgery

MI surgery involved a small incision, a three-dimensional wedge osteotomy using Shannon bur, and stabilisation with a magnesium or titanium screw (Fig. 2). Pre- and post-operative X-rays are depicted in Fig. 3. Lateral release was performed using beaver knife. A single senior surgeon performed all procedures to ensure consistency (Fig. 2). Subsequently, the wound was closed.

### Statistical analysis

Primary outcomes (AOFAS score changes) were analysed using t tests for independent samples, while secondary outcomes (IMA and HVA changes) were analysed exploratorily. The primary outcome was analysed using a t test for independent samples at a significance level of 0.05. Cohen's d is calculated to interpret the strength of the effect [12]. The following reference values help classify the effect size: *d* > 0.2 = small effect, *d* > 0.5 = moderate effect, and *d* > 0.8 = strong effect.

Baseline demographics and comorbidities were compared using t tests, Pearson’s Chi-square, and Fisher’s exact tests. The statistical analysis was performed using SPSS version 28 (IBM, Armonk, NY, USA).

## Results

### Baseline characteristics

Patients in the MI group were significantly older (*p* = 0.0024) than those in the open surgery group. Both groups had similar distributions of gender and obesity (according to the WHO definition, the threshold for obesity is defined here as a body mass index of > = 30). Still, the MI group had more pre-existing conditions (Table 1).

**Table 1:** Patients’ baseline characteristics

Variable	MI surgery (*N* = 52)	Open surgery (*N* = 51)	*p* value MI versus open	Total (*N* = 103)
Age, Average ± SD [Range]	62.0 ± 13.3 [24–80]	53.9 ± 12.9 [22–77]	0.024*	58.0 ± 13.6 [22–80]
Gender, n (%)			1.000	
Female	48 (92.3)	47 (92.2)		95 (92.2)
Male	4 (7.7)	4 (7.8)		8 (7.8)
Obese, n (%)	4 (7.7)	6 (11.8)	0.526	10 (9.7)
Deformities			0.002*	
Splayfoot, Pes plano valgus	44 (84.6)	46 (90.2)		90 (87.4)
Splayfoot, Pes plano valgus, Small toe deformities	7 (13.5)	0 (0.0)		7 (6.8)
Splayfoot	1 (1.9)	5 (9.8)		6 (5.8)
Pre-existing conditions^#^, n (%)	34 (65.4)	23 (45.1)	0.038*	57 (55.3)
Reason for surgery			0.012*	
Pain, Mobility limitations	21 (40.4)	24 (47.1)		45 (43.7)
Pain	20 (38.5)	18 (35.3)		38 (36.9)
Mobility limitations	1 (1.9)	5 (9.8)		6 (5.8)
Pain, Cosmetics	4 (7.7)	2 (3.9)		6 (5.8)
Pain, Cosmetics, Mobility Limitations	4 (7.7)	2 (3.9)		6 (5.8)
Cosmetics, Mobility limitations	2 (3.8)	0 (0.0)		2 (1.9)

*MI *minimally invasive, *SD *standard deviation, *N *number of patients, % proportion of patients. *p < 0.05, ^#^considered were diabetes mellitus type II, arterial hypertension, coronary heart disease, rheumatism

Diabetes mellitus type II, arterial hypertension, coronary heart disease, and rheumatism were more prevalent in patients in the MI group, with no significant difference between both groups. Pain was the main reason for surgery in both groups, frequently combined with mobility limitations. The average time between surgery and follow-up was 382 (± 222 SD) days. The follow-up time differed only slightly between both groups (open surgery group: 365 ± 154 vs. MI group: 399 ± 271; *p* = 0.44).

### Primary outcome

**Fig. 4:**
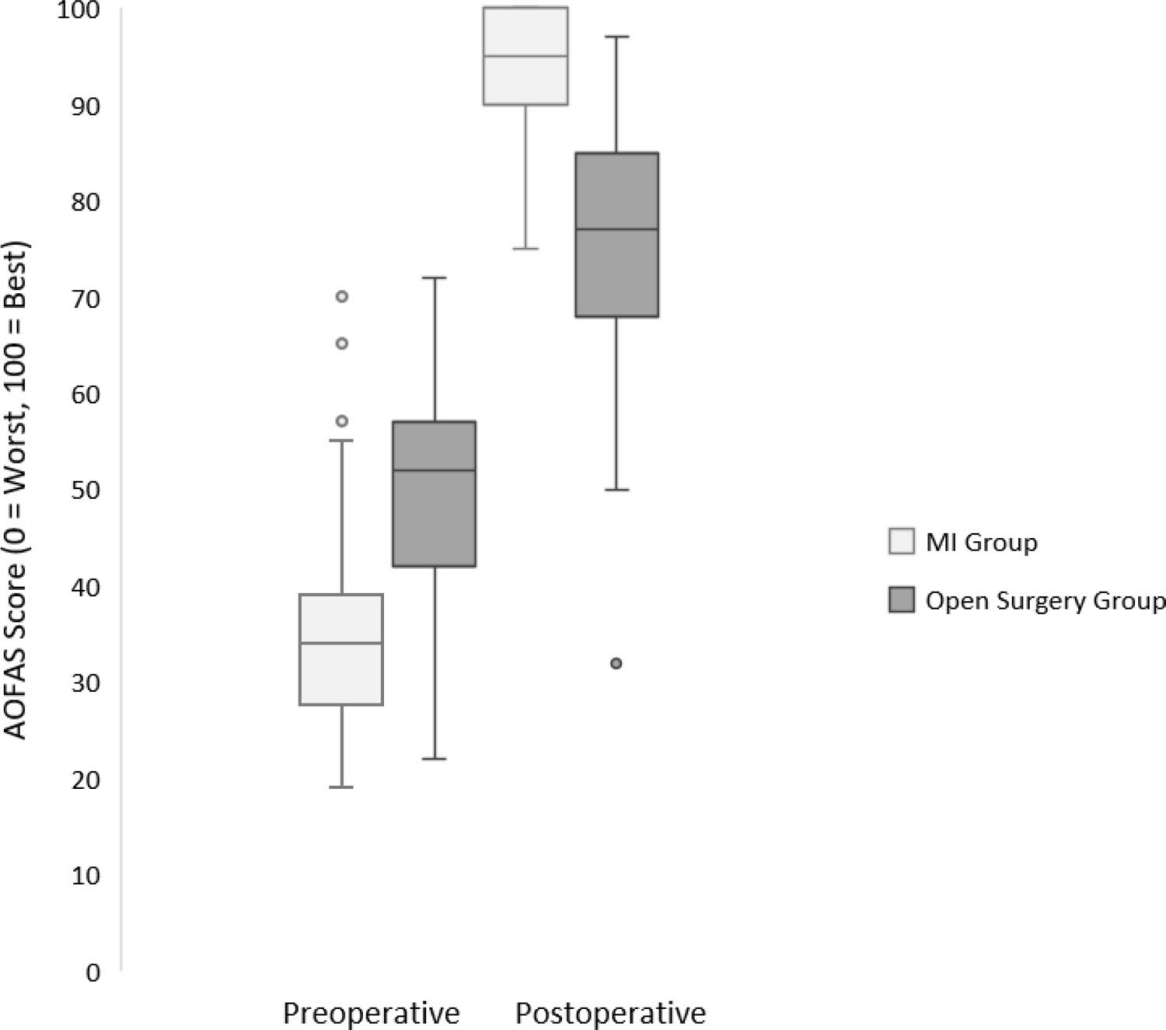
AOFAS scores—MI group versus open surgery group

MI surgery patients showed significantly more significant improvements in AOFAS scores compared to the open surgery group (*t* test: *t* = 12.454, df = 101, *p* < 0.001). The mean difference in change from baseline scores was 33.624 (95% confidence interval: 28.268–38.980), with a mean difference in the MI group of 60.2 ( ± 12.1 SD) compared to a mean difference in the open surgery group of 26.5 (± 15.1 SD), which corresponds to a large effect (Cohen’s *d* = 2.453, 95% CI: 1.941–2.964). The baseline FFI scores were lower in the MI group, but post-operative scores were significantly higher. To ensure that differing baseline scores do not confound the analysis of AOFAS change from baseline scores, post-operative values were also compared using an independent samples *t* test, yielding significant results (*p* < 0.001). The average post-operative AOFAS scores were significantly higher in patients after MI surgery (MI: 94.5 ± 6.9 SD vs. open: 75.9 ± 13.4 SD, see Table 2 and Fig. 4).

**Table 2:** AOFAS scores—MI group versus open surgery group

Variable	MI surgery (*N* = 52)			Open surgery (*N* = 51)			*p* value MI versus open
	Mean	SD	Range	Mean	SD	Range	
AOFAS score baseline	34.3	11.3	19–70	49.4	12.1	22–72	< 0.0001
AOFAS score post-operative	94.5	6.9	75–100	75.9	13.4	32–97	< 0.0001
AOFAS score change from baseline	60.2	12.0	30–81	26.5	15.0	3–66	< 0.0001

*MI* minimally invasive, *SD *standard deviation, *N *number of patients

### Subgroup analysis by IM angle deformity

In the open surgery group, 31 (60.8%) patients had an initial IM angle deformity of less than 20 degrees and 20 (39.2%) patients had an initial IM angle deformity of greater than 20 degrees. There were 9 (17.6%) patients with an initial IM angle of < 20 degrees versus 42 (82.4%) patients with an initial IM angle of > = 20 degrees in the MI group.

In the group of patients with an initial IM angle of < 20 degrees, the mean difference in change from baseline scores was 33.638 (95% confidence interval: 29.027–38.249), with a mean difference in the MI group of 56.4 (± 10.8 SD) compared to a mean difference in the open surgery group of 22.8 (± 12.7 SD), which corresponds to a large effect (Cohen’s d = 2.852, 95% CI: 2.304–3.401). In the group of patients with more severe IM angle deformity, the mean difference in change from baseline scores was 28.630 (95% confidence interval: (23.174–34.087), with a mean difference in the MI group of 60.9 (± 12.1 SD) compared to a mean difference in the open surgery group of 32.3 (± 16.4 SD), which corresponds to a large effect (Cohen’s *d *= 1.993, 95% CI: 1.533–2.452)).

### Secondary outcomes

**Fig. 5:**
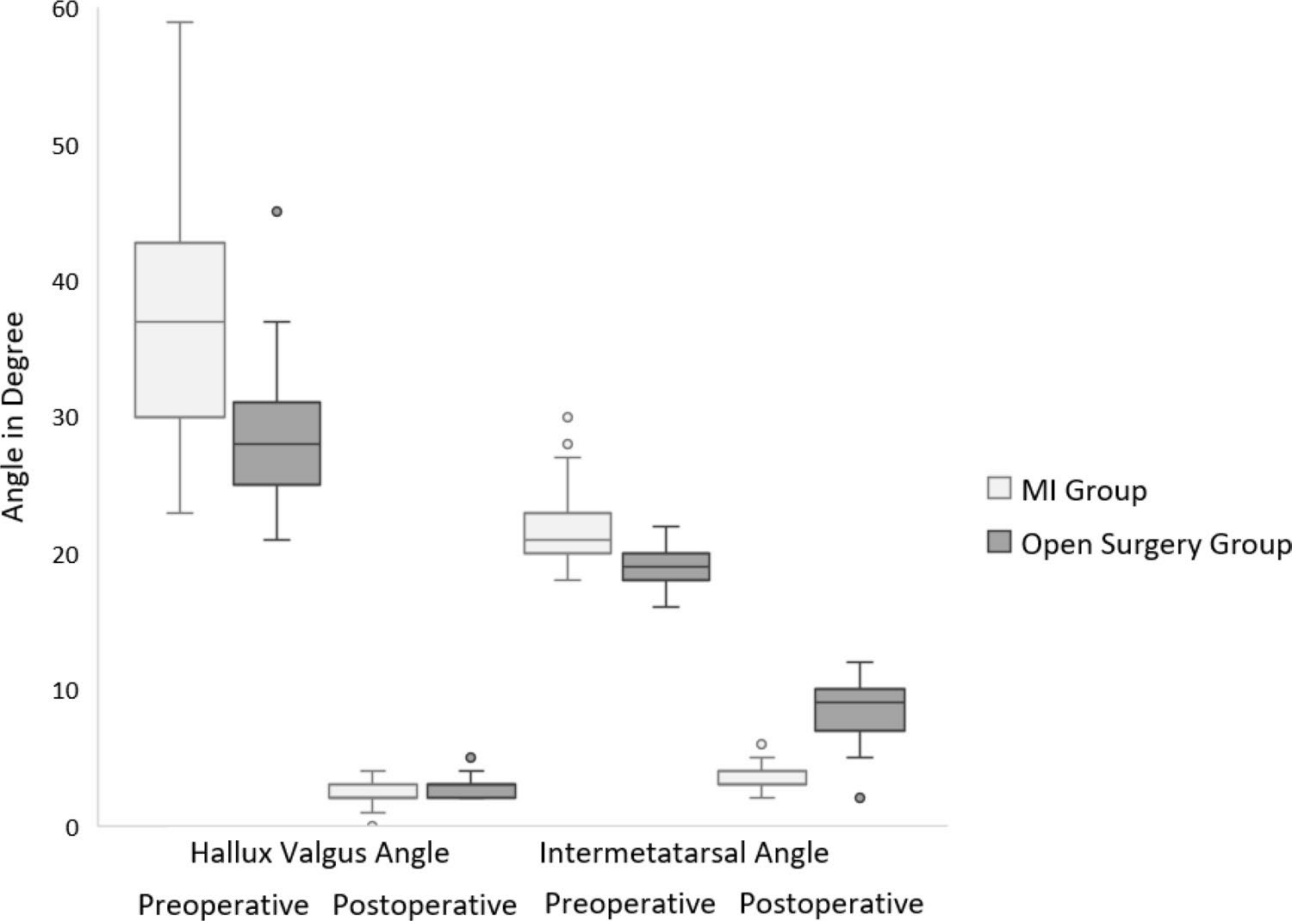
HV angle and IM angle—MI group versus open surgery group

Post-operative IMA was significantly lower in the MI group (3.1 ± 0.9° vs. 8.8 ± 2.2°, *p* < 0.001), while HVA correction was similar between groups (Fig. 5). As for post-operative HVA, it averaged 2.1 degrees (± 0.8 SD) for minimally invasive surgery and 2.8 degrees (± 0.9 SD) for open surgery. The difference between the two procedures was not statistically significant (*p* = 0.120). Preoperatively, IMA averaged 22.0 degrees (± 2.9 SD) for MI surgery and 19.1 degrees (± 1.4 SD) for open surgery, and HVA averaged 37.5 degrees (± 8.9 SD) for MI surgery and 28.2 degrees (± 4.3 SD) for open surgery. Thus, both preoperative angles were slightly larger on average in the MI group than in the open surgery group. Accordingly, the reductions in both angles between preoperative and post-operative values were significantly greater for MI surgery patients (*p* < 0.001 for both IMA and HVA). The complication rate was significantly lower in the MI group (3.8% vs. 33.3%, *p* < 0.001). Surgery duration and hospital stay were also shorter in the MI group.

While only two patients (3.8%) reported one or more complications in the MI group, 17 patients (33.3%) suffered from one or more complications in the open surgery group, resulting in a significantly lower complication rate in the MI group (*p* < 0.001).

Only two cases of persistent swelling were documented after MI surgery. Following open surgery, persistent swelling was noted in nine patients (17.7%), five (9.8%) patients complained of continuous pain, and four (7.8%) had wound-healing complications. Pseudarthrosis, infection, material breakage, and the need for revision surgery occurred in a maximum of two cases.

### Pain, functionality, satisfaction, length of hospital stay, and surgery duration

Preoperative pain levels were similar between both groups (MI: 3.2, open: 2.9). Post-operatively, pain levels reduced significantly (MI: 1.2, open: 1.9). Functionality scores showed no significant preoperative differences (MI: 2.7, open: 2.6) but improved post-operatively (MI: 1.2, open: 1.8).

Patient satisfaction was higher in the MI group (MI: 1.7, open: 2.3). Surgeries were shorter for the MI group (19.7 ± 2.3 min vs. 80.7 ± 6.8 min), and hospital stays were shorter (MI: 0.9 ± 0.3 days vs. open: 2.0 ± 0.0 days).

## Discussion

Minimally invasive (MI) techniques for HV correction have gained popularity due to advantages like limited tissue dissection, smaller wounds, reduced operation time, and lower costs [13]. Despite over 150 surgical procedures described, high-evidence prospective studies are scarce, and the optimal treatment modality remains controversial [14–17].

This study used a third-generation MI technique involving proximal osteotomy with a stable bicortical fixation using a titanium screw, as described by Brogan et al. [5]. Previous techniques, like the Reverdin-Isham osteotomy without fixation, had high complication rates leading to abandonment in favour of more stable screw fixations [18–20]. Therefore, the authors recommend stable bicortical screw fixation instead of K-Wire fixation.

This study evaluated patient outcomes after MI or open HV correction without accompanying deformities, including pain, functionality, and patient satisfaction.

MI surgery showed better overall clinical outcomes, with significantly higher post-operative AOFAS scores (MI: 94.5 ± 6.9 vs. open: 75.9 ± 13.4). This improvement comprised all individual AOFAS items: pain, functionality, and patient satisfaction. Even if there were significant differences between the two groups in preoperative parameters that may influence patient outcomes, biased results are very unlikely. Despite more severe preoperative conditions in the MI group, both the post-operative and the change from baseline AOFAS scores were clearly superior to the values in the open group.

Our results align with a meta-analysis by Ji et al., which found higher AOFAS scores and less post-operative pain for MI procedures. The authors conclude that MI procedures were more effective in the treatment of HV and achieved better radiological and clinical outcomes [15]. Lu et al. had similar findings, though without significant AOFAS improvements [16, 21].

Conversely, Kaufmann et al. reported no significant differences between MI and open chevron osteotomy regarding pain, AOFAS scores, radiographic outcomes, range of motion, or patient satisfaction [16]. Singh et al. concluded that while MI procedures provided satisfactory results for moderate HV cases, open surgery yielded better functional outcomes [22]. It is worth mentioning that the follow-up duration between these studies is extremely variable, ranging from 6 months to 8 years, and differing surgical techniques, which, in our view, limit the interpretation of meta-analyses in HV surgery [22–24]. Similarly, Alimy et al. [1] did not identify significant advantages of MI surgery over open procedures. However, they did observe a discrete benefit of MI surgery in terms of post-operative pain and cosmetic results [25].

Interestingly, our study showed a significantly lower complication rate for MI surgery (3.8% vs. 33.3%, *p* < 0.001). Complications in HV correction, such as under-correction, overcorrection, nonunion, malunion, avascular necrosis, infection, nerve injury, and patient dissatisfaction, occur in 10–55% of cases [26, 27]. The three-dimensional osteotomy technique used in MI surgery reduces recurrence and metatarsal misload [28, 29].

Concerning functionality, operation time, and hospital stay the present study was also in line with previous literature [22, 30, 31].

Another interesting finding of the current study is a significantly reduced operation time (19.7 ± 2.3 min vs. 80.7 ± 6.8 min). Time-consuming tissue dissection can be omitted using MI techniques. Furthermore, hospital stay was significantly shorter (0.9 ± 0.3 days vs. 2.0 ± 0.0 days) in the MI surgery group. Similar results have been reported in the literature and favour MI techniques over open surgery.

### Radiological outcome

Radiologically, MI surgery was more effective in restoring IMA, with no significant difference in HVA correction between groups. No cases of nonunion, osteonecrosis, or early recurrence were observed. Kaufmann et al. found similar IMA and HVA reductions in both groups, while Vieira Cardoso et al. reported better IMA reduction with open surgery. This discrepancy could be due to the learning curve associated with MI techniques [16, 20]. Unlike Chan et al., we did not observe a discrepancy between clinical outcomes and angle measurements. However, clinical and radiological outcomes are generally known not to have a linear correlation [32, 33].

While the operative technique in the mentioned study from Kadakia et al. involved a fixation with K-wires, a more stable fixation can be achieved using a titanium screw. It should also be noted that the authors recommend a bicortical screw fixation.

### Limitations

The current study is limited by the inherited deficiencies of a prospective study and a short follow-up time. In addition, we acknowledged that surgery was performed at one single centre and carried out by a single senior surgeon specialising in foot and ankle surgery. To confirm the encouraging results of the current study, large-scale randomised controlled trials with sufficient follow-up are needed.

## Conclusion

This study compared open and MI HV correction procedures, finding that MI surgery resulted in better pain reduction, functionality, patient satisfaction, lower complication rates, greater IMA correction, reduced operation times, and shorter hospital stays. Further randomised controlled trials are necessary for broader adoption and verification of these results.

## References

[1] Cai Y, Song Y, He M et al (2023) Global prevalence and incidence of hallux valgus: a systematic review and meta-analysis. J Foot Ankle Res 16(1):63 37726760 10.1186/s13047-023-00661-9 PMC10510234

[2] Coughlin MJ, Jones CP (2007) Hallux valgus: demographics, etiology, and radiographic assessment. Foot Ankle Int 28(7):759–777 17666168 10.3113/FAI.2007.0759

[3] Nix S, Smith M, Vicenzino B (2010) Prevalence of hallux valgus in the general population: a systematic review and meta-analysis. J Foot Ankl Res 3:21 10.1186/1757-1146-3-21 PMC2955707 20868524

[4] Roddy E, Zhang W, Doherty M (2008) Prevalence and associations of hallux valgus in a primary care population. Arthritis Care Res 59(6):857–862 10.1002/art.23709 18512715

[5] Brogan K, Voller T, Gee C, Borbely T, Palmer S (2014) Third-generation minimally invasive correction of hallux valgus: technique and early outcomes. Int Orthop 38(10):2115–2121 25128969 10.1007/s00264-014-2500-1

[6] Wulker N, Mittag F (2012) The treatment of hallux valgus. Dtsch Arztebl Int 109(49):857–867 23267411 10.3238/arztebl.2012.0857 PMC3528062

[7] Cohena-Jimenez M, Prieto-Dominguez R, Perez-Belloso AJ, Muriel-Sanchez JM, Gomez-Carrion A, Montano-Jimenez P (2023) Comparison of resorbable and non-resorbable osteosynthesis material in hallux surgery: a systematic review. Life (Basel) 13(10):2018 37895399 10.3390/life13102018 PMC10608219

[8] He T, Zhou L, Sun Y (2024) Letter to the editor: does minimally invasive surgery provide better clinical or radiographic outcomes than open surgery in the treatment of hallux valgus deformity? A systematic review and meta-analysis. Clin Orthop Relat Res® 482(1):213–214 37815373 10.1097/CORR.0000000000002890 PMC10723868

[9] Kipping R (2018) Operation Fuß: fragen an den spezialisten. Springer, Berlin

[10] Maffulli N, Longo UG, Marinozzi A, Denaro V (2011) Hallux valgus: effectiveness and safety of minimally invasive surgery. A syst Rev Br Med Bull 97:149–167 10.1093/bmb/ldq027 20710024

[11] Trnka HJ, Krenn S, Schuh R (2013) Minimally invasive hallux valgus surgery: a critical review of the evidence. Int Orthop 37(9):1731–1735 23989262 10.1007/s00264-013-2077-0 PMC3764277

[12] Cohen J (1988) Statistical power analysis for the behavioral sciences. L. Erlbaum Associates, Hillsdale

[13] Palmanovich E, Ohana N, Tavdi A et al (2023) A modified minimally invasive osteotomy for hallux valgus enables reduction of malpositioned sesamoid bones. Arch Orthop Trauma Surg 143(10):6105–6112 37202550 10.1007/s00402-023-04868-0

[14] Gonzalez TA, Smith JT, Bluman EM, Ready LV, Ciurylo W, Chiodo CP (2018) Treatment of hallux valgus deformity using a suture button device: a preliminary report. Foot Ankl Orthop 3(4):247301141880695 10.1177/2473011418806951 PMC10467187 37655934

[15] Ji L, Wang K, Ding S, Sun C, Sun S, Zhang M (2022) Minimally invasive vs. open surgery for hallux valgus: a meta-analysis. Front Surg 9:843410 35388365 10.3389/fsurg.2022.843410 PMC8978717

[16] Kaufmann G, Dammerer D, Heyenbrock F, Braito M, Moertlbauer L, Liebensteiner M (2019) Minimally invasive versus open chevron osteotomy for hallux valgus correction: a randomized controlled trial. Int Orthop 43(2):343–350 29869014 10.1007/s00264-018-4006-8 PMC6399198

[17] Trnka HJ (2021) Percutaneous, MIS and open hallux valgus surgery. EFORT Open Rev 6(6):432–438 34267933 10.1302/2058-5241.6.210029 PMC8246107

[18] Bauer T, Biau D, Lortat-Jacob A, Hardy P (2010) Percutaneous hallux valgus correction using the Reverdin-Isham osteotomy. Orthop Traumatol Surg Res 96(4):407–416 20488776 10.1016/j.otsr.2010.01.007

[19] Bösch P, Wanke S, Legenstein R (2000) Hallux valgus correction by the method of Bösch: a new technique with a seven-to-ten-year follow-up. Foot Ankle Clin 5(3):485–498 11232393

[20] Jowett CRJ, Bedi HS (2017) Preliminary results and learning curve of the minimally invasive chevron akin operation for hallux valgus. J Foot Ankl Surg 56(3):445–452 10.1053/j.jfas.2017.01.002 28237566

[21] Lu J, Zhao H, Liang X, Ma Q (2020) Comparison of Minimally Invasive and Traditionally Open Surgeries in Correction of Hallux Valgus: A Meta-Analysis. J Foot Ankl Surg 59(4):801–806 10.1053/j.jfas.2019.03.021 32600562

[22] Singh MS, Khurana A, Kapoor D, Katekar S, Kumar A, Vishwakarma G (2020) Minimally invasive vs open distal metatarsal osteotomy for hallux valgus - A systematic review and meta-analysis. J Clin Orthop Trauma 11(3):348–356 32405192 10.1016/j.jcot.2020.04.016 PMC7211908

[23] Choi WJ, Yoon HK, Yoon HS, Kim BS, Lee JW (2009) Comparison of the proximal chevron and Ludloff osteotomies for the correction of hallux valgus. Foot Ankle Int 30(12):1154–1160 20003873 10.3113/FAI.2009.1154

[24] Giannini S, Cavallo M, Faldini C, Luciani D, Vannini F (2013) The SERI distal metatarsal osteotomy and Scarf osteotomy provide similar correction of hallux valgus. Clin Orthop Relat Res 471(7):2305–2311 23494184 10.1007/s11999-013-2912-z PMC3676577

[25] Alimy AR, Polzer H, Ocokoljic A et al (2023) Does Minimally Invasive Surgery provide better clinical or radiographic outcomes than open surgery in the treatment of hallux valgus deformity? A systematic review and meta-analysis. Clin Orthop Relat Res® 481(6):1143–1155 36332131 10.1097/CORR.0000000000002471 PMC10194698

[26] Filippi J, Briceno J (2020) Complications after metatarsal osteotomies for hallux valgus: malunion, nonunion, avascular necrosis, and metatarsophalangeal osteoarthritis. Foot Ankle Clin 25(1):169–182 31997743 10.1016/j.fcl.2019.10.008

[27] Monteagudo M, Martínez-de-Albornoz P (2020) Management of complications after hallux valgus reconstruction. Foot Ankl Clin 25(1):151–167 10.1016/j.fcl.2019.10.011 31997742

[28] Iyer S, Demetracopoulos CA, Sofka CM, Ellis SJ (2015) High rate of recurrence following proximal medial opening wedge osteotomy for correction of moderate hallux valgus. Foot Ankle Int 36(7):756–763 25780267 10.1177/1071100715577195

[29] Rodríguez-Reyes G, López-Gavito E, Pérez-Sanpablo AI et al (2014) Dynamic plantar pressure distribution after percutaneous hallux valgus correction using the Reverdin-Isham osteotomy. Rev Invest Clin 66(Suppl 1):S79-84 25264801

[30] Torrent J, Baduell A, Vega J, Malagelada F, Luna R, Rabat E (2021) Open vs minimally invasive scarf osteotomy for hallux valgus correction: a randomized controlled trial. Foot Ankl Int 42(8):982–993 10.1177/10711007211003565 34024185

[31] Vieira Cardoso D, Veljkovic A, Wing K, Penner M, Gagne O, Younger A (2022) Cohort comparison of radiographic correction and complications between minimal invasive and open lapidus procedures for hallux valgus. Foot Ankl Int 43(10):1277–1284 10.1177/10711007221112088 PMC9527364 35880322

[32] Chan CX, Gan JZ, Chong HC, Rikhraj Singh I, Ng SYC, Koo K (2019) Two year outcomes of minimally invasive hallux valgus surgery. Foot Ankle Surg 25(2):119–126 29409293 10.1016/j.fas.2017.09.007

[33] Thordarson D, Ebramzadeh E, Moorthy M, Lee J, Rudicel S (2005) Correlation of hallux valgus surgical outcome with AOFAS forefoot score and radiological parameters. Foot Ankl Int 26(2):122–127 10.1177/107110070502600202 15737253

